# Anti-POSTN and Anti-TIMP1 Autoantibodies as Diagnostic Markers in Esophageal Squamous Cell Carcinoma

**DOI:** 10.3389/fgene.2022.860611

**Published:** 2022-04-26

**Authors:** Weihong Xie, Guiying Sun, Jicun Zhu, Huimin Wang, Zhuo Han, Peng Wang

**Affiliations:** ^1^ Department of Oral and Maxillofacial Surgery, The First Affiliated Hospital, Zhengzhou University, Zhengzhou, China; ^2^ Department of Epidemiology and Statistics and State Key Laboratory of Esophageal Cancer Prevention & Treatment, College of Public Health, Zhengzhou University, Zhengzhou, China; ^3^ Henan Key Laboratory of Tumor Epidemiology and State Key Laboratory of Esophageal Cancer Prevention & Treatment, Zhengzhou University, Zhengzhou, China; ^4^ Academy of Medical Science, Zhengzhou University, Zhengzhou, China

**Keywords:** autoantibody, POSTN, TIMP1, immunodiagnosis, esophageal squamous cell carcinoma, biomarker

## Abstract

Esophageal cancer is one of the most commonly diagnosed malignant gastrointestinal tumors. The aim of the study was to explore the diagnostic values of anti-POSTN and anti-TIMP1 autoantibodies in esophageal squamous cell carcinoma (ESCC). Differentially expressed genes (DEGs) associated with esophageal cancer were screened out by the LIMMA method in the Gene Expression Profiling Interactive Analysis (GEPIA) platform. Search Tool for the Retrieval of Interacting Genes (STRING) was used to construct the protein–protein interaction (PPI) based on highly DEGs. The candidate hub genes were the intersection genes calculated based on degree and Maximal Clique Centrality (MCC) algorithms *via* Cytoscape. A total of 370 participants including 185 ESCC patients and 185 matched normal controls were enrolled in enzyme-linked immunosorbent assay (ELISA) to detect the expression levels of autoantibodies corresponding to POSTN and TIMP1 proteins. A total of 375 DEGs with high expression were obtained in esophageal cancer. A total of 20 hub genes were acquired using the cytoHubba plugin by degree and MCC algorithms. The expression levels of anti-POSTN and anti-TIMP1 autoantibodies were higher in the sera of ESCC patients (*p* < 0.05). Anti-POSTN autoantibody can diagnose ESCC patients with an AUC of 0.638 at the specificity of 90.27% and sensitivity of 27.57%, and anti-TIMP1 autoantibody can diagnose ESCC patients with an AUC of 0.585 at the specificity of 90.27% and sensitivity of 20.54% (*p* < 0.05). In addition, anti-POSTN and anti-TIMP1 autoantibodies can distinguish ESCC patients from normal controls in most clinical subgroups (*p* < 0.05). In conclusion, anti-POSTN and anti-TIMP1 autoantibodies may be considered the potential biomarkers in the clinical diagnosis of ESCC.

## Introduction

Esophageal cancer (EC) is one of the most common malignant tumors of the digestive tract and ranks sixth among malignant tumors in mortality worldwide (Sung et al., 2021). GLOBOCAN 2020 shows that there are 604,100 newly diagnosed EC cases worldwide and 544,076 people died in the same period (Sung et al., 2021). Many patients are in the advanced stage at the initial diagnosis and have poor prognosis because of the occult onset and no obvious early symptoms (Li et al., 2021). The 5-year survival rate can reach as high as 60%, if patients with EC could be diagnosed early and treated with surgery (He et al., 2021). At present, the commonly used clinical methods for diagnosing EC include endoscopy, CT scan, barium meal examination, and pathological biopsy, but they are expensive and invasive and cannot be used for screening (Guo et al., 2018). Traditional tumor serological markers such as cancer antigen 12-5 (CA12-5), carcinoembryonic antigen (CEA), squamous cell carcinoma antigen (SCCA), and carbohydrate antigen199 (CA19-9) are used as auxiliary markers of clinical diagnosis for EC, but the sensitivity and specificity of these markers are poor (Kosugi et al., 2004; Mroczko et al., 2008). Therefore, it is of great clinical value to explore better diagnostic markers of EC.

Bioinformatics can conduct in-depth analysis of open biological databases such as tissues, cell genes, and proteins, and provide a potential theoretical basis for cancer early diagnosis and treatment (Wang Y. et al., 2021). Many scholars have used the database to analyze differentially expressed genes (DEGs) in the progression of lung cancer, gastric cancer, breast cancer, and other tumors (Ren et al., 2020; Wang H. et al., 2021; Shan et al., 2021). The proteins encoded by these DEGs could be used as molecular markers for tumor diagnosis and prognosis (Zhao et al., 2021). Moreover, autoantibodies against tumor-associated antigens (TAAbs) can exist stably in the serum of cancer patients and can be detected months or even years before the onset of clinical symptoms (Tan and Zhang, 2008). Therefore, they have the potential to be biomarkers for early immunodiagnosis of cancers. Many studies have reported higher levels of TAAbs in the serum of patients with cancer, such as hepatocellular carcinoma, ovarian cancer, breast cancer, and esophageal cancer (Katchman et al., 2017; Xu and Liu, 2017; Loke and Lee, 2018; Zheng et al., 2018). At present, there is no recognized marker for the detection of esophageal cancer, and the aim of the present study was to identify novel TAAbs to improve the sensitivity and specificity.

In China, esophageal squamous cell carcinoma (ESCC) patients account for more than 90% of esophageal cancer patients (Cao and Sun, 2016). In this study, we analyzed the EC-related data from TCGA (The Cancer Genome Atlas) and GTEx (the Genotype-Tissue Expression) databases to identify the differentially expressed genes in esophageal cancer. Then, we further attained the hub genes in the highly expressed differential genes *via* degree and Maximal Clique Centrality (MCC) algorithms, and the proteins encoded by them were regarded as candidate TAAs. Finally, enzyme-linked immunosorbent assay (ELISA) was used to evaluate the diagnostic value of the corresponding autoantibodies of TAAs for ESCC.

## Materials and Methods

### Screening of Candidate Hub Genes

Based on TCGA (The Cancer Genome Atlas) and GTEx (the Genotype-Tissue Expression) databases, we used the GEPIA (http://gepia2.cancer-pku.cn/#degenes) web server to screen DEGs associated with esophageal cancer by the LIMMA method (Tang et al., 2019). DEGs satisfying the criteria with adjusted *p* < 0.01 and |log2 fold change (FC)|>2 were designated as statistically significant. The volcano map of DEGs was drawn using the SangerBox tools, a free online platform for data analysis (http://www.sangerbox.com/tool).

STRING (Search Tool for the Retrieval of Interacting Genes, https://cn.string-db.org/) was adopted to construct the protein–protein interaction (PPI) based on highly DEGs (Franceschini et al., 2013). The cytoHubba plug-in Cytoscape (version 3.8.2) was used to calculate hub nodes to select the top 20 DEGs (Shannon et al., 2003). The intersection genes calculated based on degree and MCC algorithms were used as the candidate hub genes.

### Enzyme-Linked Immunosorbent Assay

Through bioinformatics methods, we know that POSTN and TIMP1 were highly expressed in patients with esophageal cancer. Therefore, we further detect the expression levels of autoantibodies corresponding to these two proteins in the serum of patients with ESCC by ELISA. Purified recombinant proteins POSTN and TIMP1 were purchased from the CLOUD-CLONE CORP (Wuhan, China). Horseradish peroxidase (HRP)-conjugated mouse anti-human IgG (Wuhan Aoko Biotechnology Co. Ltd.) was used as the secondary antibody. The coated concentrations of POSTN and TIMP1 proteins were both 0.5 ng/ml. Each ELISA plate included six repeated serum samples and two blank controls. The repeated serum samples were used to normalize the difference between plates, and the blank controls were consulted for quality control. The detailed steps of ELISA were described in our previous study (Wang et al., 2018). The microplate reader was performed to measure the optical density (OD) of wells at 450 and 620 nm. The absorbance difference between 450 and 620 nm was used for the subsequent analysis.

### Study Participants

The serum samples of 185 patients with ESCC included in this study came from a third class hospital in Henan Province. They were all patients with ESCC diagnosed by histopathology and without any treatment and did not suffer from other cancer diseases. The serum samples of 185 normal controls were received from the specimen bank of Henan Key Laboratory of Tumor Epidemiology. The normal controls excluded autoimmune diseases, esophageal cancer, and related diseases.

### Statistical Analysis

IBM SPSS 22.0 and GraphPad Prism 9.1.1 were used in the study. All statistical analyses were based on the two-tailed test, and *p* < 0.05 was considered to be statistically significant. The non-parametric test was adopted to compare the expression levels of autoantibodies between ESCC patients and normal controls. The OD value corresponding to the maximum Youden’s index when the specificity is greater than 90% was determined as the cutoff value. The Chi-square test was employed to analyze the positive rates of autoantibodies in different clinical subgroups in all ESCC patients. The receiver operating characteristic curve (ROC) was used to evaluate the diagnostic value of the autoantibodies in different groups. The positive likelihood ratio (PLR), negative likelihood ratio (NLR), positive predictive value (PPV), negative predictive value (NPV), accuracy, and Youden’s index were calculated to estimate the diagnostic value of the two autoantibodies.

## Results

### Identified Potential Hub Genes

**FIGURE 1:**
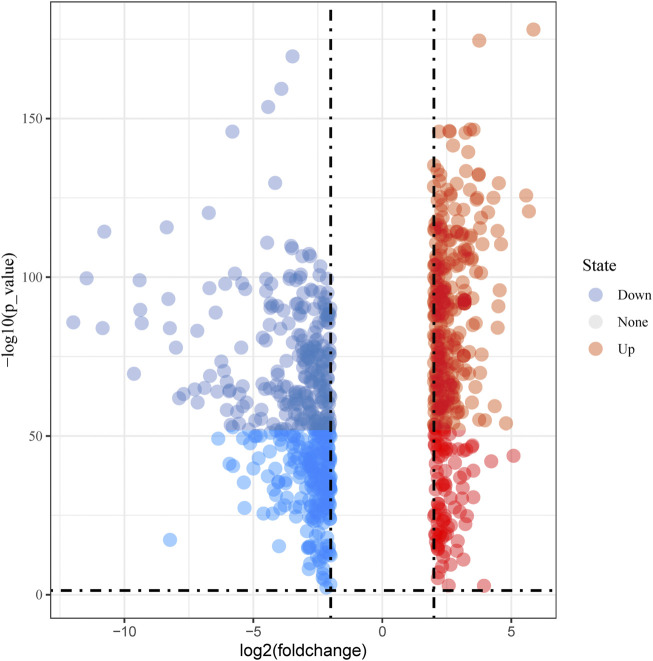
Volcano plot of differentially expressed genes.

**FIGURE 2:**
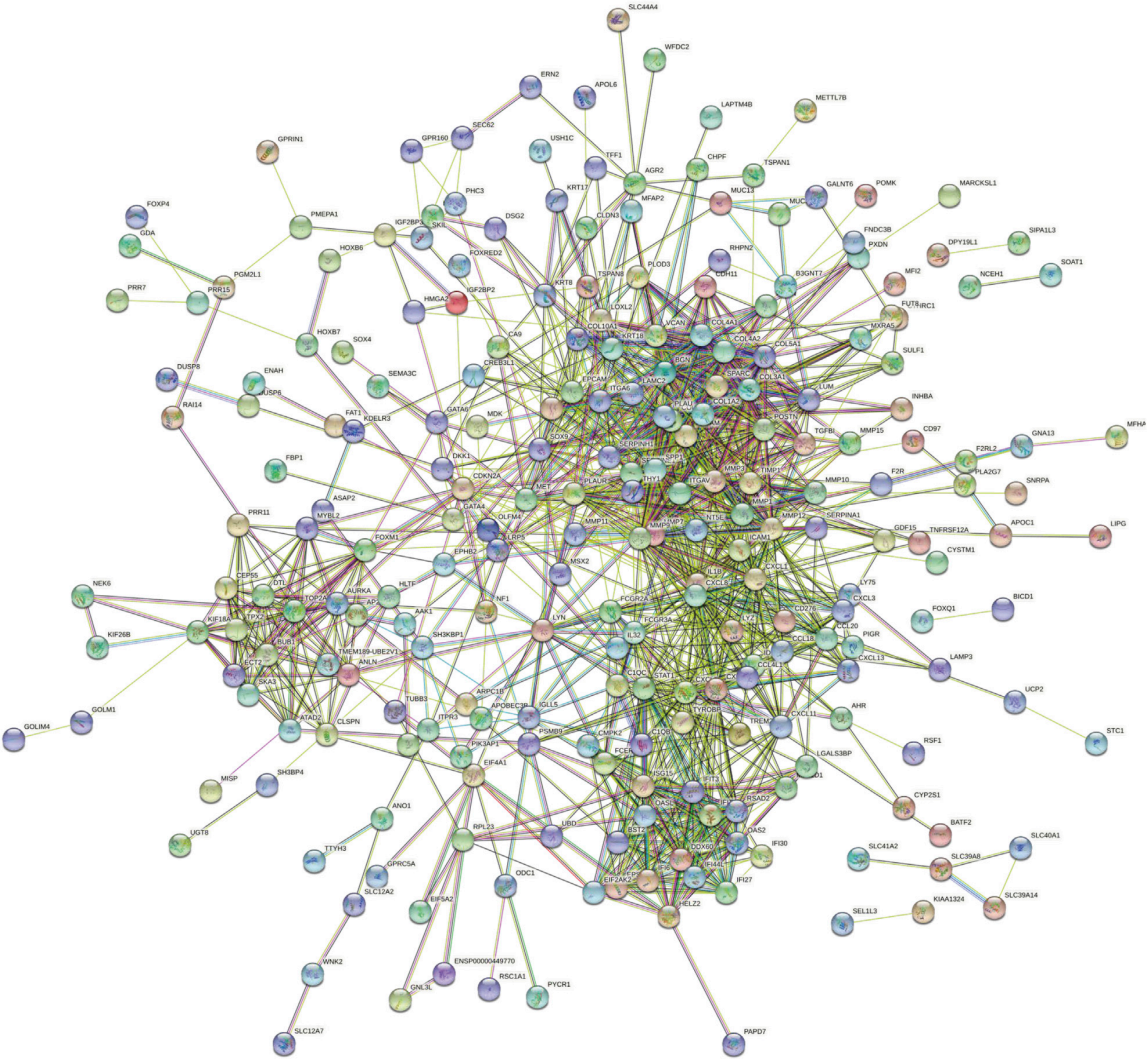
PPI network for highly DEGs. PPI, protein–protein interaction; DEGs, differentially expressed genes.

**FIGURE 3:**
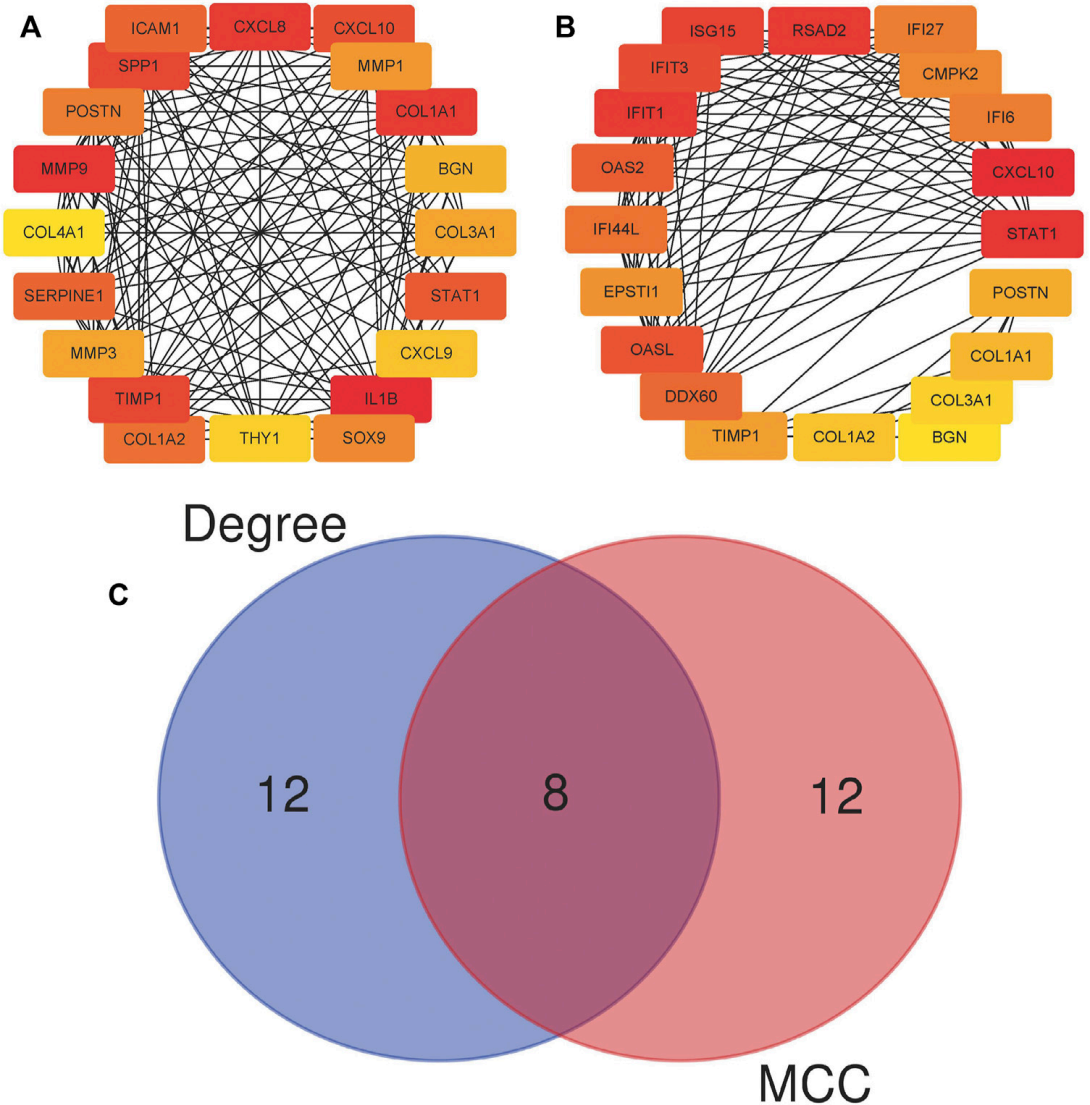
Twenty hub genes calculated using the cytoHubba plugin. **(A)** Twenty hub genes based on the degree algorithm. **(B)** Twenty hub genes based on the MCC algorithm; MCC, Maximal Clique Centrality. **(C)** Wien diagram of hub genes attained from two algorithms.

By analyzing the gene expression profiles of esophageal cancer tissues and adjacent tissues, the differentially expressed genes in esophageal cancer tissues were significantly higher or lower than those in normal esophageal tissues. After differential analysis, a total of 375 DEGs with high expression and 449 DEGs with low expression were obtained in esophageal cancer, and the results of the visualization are in a volcano plot (Figure 1). The PPI network generated by STRING is presented in Figure 2 for highly DEGs. The 20 hub genes calculated using the cytoHubba plugin were IL1B, MMP9, CXCL8, COL1A1, SPP1, TIMP1, CXCL10, STAT1, ICAM1, SERPINE1, COL1A2, POSTN, SOX9, MMP1, MMP3, COL3A1, BGN, CXCL9, THY1, and COL4A1 by degree algorithm (Figure 3A), and the 20 hub genes calculated using the cytoHubba plugin were CXCL10, STAT1, IFIT1, RSAD2, ISG15, IFIT3, OASL, OAS2, DDX60, IFI44L, IFI27, IFI6, CMPK2, EPSTI1, TIMP1, POSTN, COL1A1, COL1A2, COL3A1, and BGN by MCC algorithm (Figure 3B).

A total of eight genes were selected by both algorithms, including CXCL10, STAT1, POSTN, TIMP1, COL1A1, COL1A2, COL3A1, and BGN (Figure 3C). According to the importance ranking of these eight genes in the two algorithms and the query of the relevant literature, we finally determined POSTN and TIMP1 as the target genes of this study. In terms of importance ranking, according to the comprehensive importance ranking calculated by the two algorithms, the order of the eight genes was CXCL10, STAT1, TIMP1, COL1A1, POSTN, COL1A2, BGN, and COL3A1. In addition, POSTN functioned as a cell adhesion molecule and participated in many biological processes, including cell adhesion, invasion, metastasis, and tumor angiogenesis (Bao et al., 2004; Kudo et al., 2006; Siriwardena et al., 2006). TIMP1 promotes the growth of human keratinocytes and several other cell types, inhibits apoptosis, and promotes growth (Gasson et al., 1985; Bertaux et al., 1991; Guedez et al., 1998). A high expression of TIMP1 has a significant correlation with a poor prognosis of cancer (Jackson et al., 2017). Therefore, the proteins encoded by POSTN and TIMP1 have the possibility of being potential tumor-associated antigens, and we further detected the level of anti-TAA autoantibodies in the subjects’ serum by ELISA experimental.

### Characteristics of Study Participants

The expression levels of anti-POSTN and anti-TIMP1 autoantibodies obtained by bioinformatics methods in ESCC patients and normal controls were verified by ELISA. Sera of 185 ESCC patients and 185 normal controls were used in ELISA. The study was approved by the Medical Ethics Committee of Zhengzhou University, and informed consent was obtained from all participants. The detailed clinical information of 370 participants is described in Table 1. There was no significant difference in gender (*p* = 0.658) and age (*p* = 0.223) between ESCC patients and normal controls.

**TABLE 1:** Characteristics of study participants.

Group	ESCC patient	Normal control
Number	185	185
Gender		
Male, *n* (%)	126 (68.11)	122 (65.95)
Female, *n* (%)	59 (31.89)	63 (34.05)
Age		
Mean age ± SD (years)	64.2 ± 8.3	64.0 ± 7.2
Age range (years)	42–88	53–93
Tumor site		
Upper thorax, *n* (%)	22 (11.89)	—
Middle thorax, *n* (%)	66 (35.68)	—
Lower thorax, *n* (%)	39 (21.08)	—
Unknown, *n* (%)	58 (31.35)	—
Family tumor history		
Yes, *n* (%)	47 (25.41)	—
No, *n* (%)	136 (73.51)	—
Unknown, *n* (%)	2 (1.08)	—
Histological grade		
High, *n* (%)	3 (1.62)	—
Medium, *n* (%)	46 (24.86)	—
Low, *n* (%)	40 (21.62)	—
Unknown, *n* (%)	96 (51.89)	—
TNM stage		
Ⅰ, *n* (%)	65 (35.14)	—
II, *n* (%)	28 (15.14)	—
III, *n* (%)	43 (23.24)	—
IV, *n* (%)	25 (13.51)	—
Unknown	24 (12.97)	—
Lymph node metastasis		
Positive, *n* (%)	73 (39.46)	—
Negative, *n* (%)	101 (54.59)	—
Unknown, *n* (%)	11 (5.95)	—
Distant metastasis		
Yes, *n* (%)	19 (10.27)	—
No, *n* (%)	118 (63.78)	—
Unknown, *n* (%)	48 (25.95)	—

ESCC, esophageal squamous cell carcinoma; SD, standard deviation.

### The Diagnostic Values of Anti-POSTN and Anti-TIMP1 Autoantibodies in Esophageal Squamous Cell Carcinoma

**FIGURE 4:**
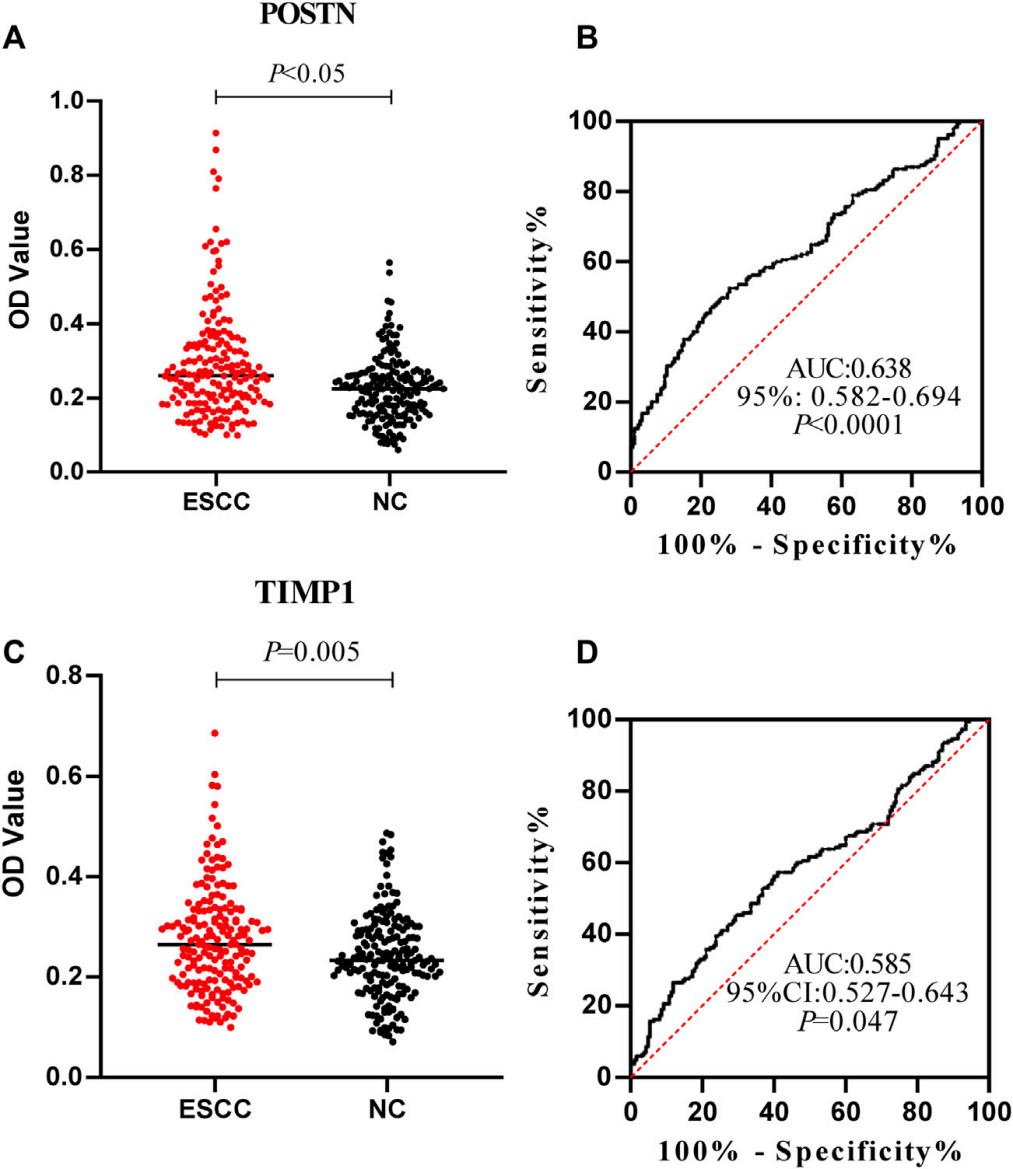
Expression level and diagnostic value of anti-POSTN and anti-TIMP1 autoantibodies in ESCC patients and normal controls. **(A,C)** Scatter plot described the expression level of anti-POSTN and anti-TIMP1 autoantibodies in the ESCC group and normal control group. **(B,D)** Receiver operating characteristic curve of anti-POSTN and anti-TIMP1 autoantibodies in diagnosing ESCC patients and normal controls. ESCC, esophageal squamous cell carcinoma; NC, normal controls.

The expression levels of anti-POSTN and anti-TIMP1 autoantibodies in ESCC patients and normal controls were detected by ELISA. The expression level of anti-POSTN autoantibody in ESCC patients was distinctly higher than that in the normal controls (mean ± SD: 0.292 ± 0.149 vs. 0.224 ± 0.087) (Figure 4A). Anti-POSTN autoantibody can diagnose ESCC patients with an AUC of 0.638 at the specificity of 90.27% and sensitivity of 27.57% (Figure 4B). The expression level of anti-TIMP1 autoantibody in ESCC patients was elevated compared with that of normal controls (mean ± SD: 0.274 ± 0.106 vs. 0.240 ± 0.085) (Figure 4C). Anti-TIMP1 autoantibody can diagnose ESCC patients with an AUC of 0.585 at the specificity of 90.27% and sensitivity of 20.54% (Figure 4D). In order to further evaluate the diagnostic values of anti-POSTN and anti-TIMP1 autoantibodies in ESCC, other diagnostic parameters were also calculated, as shown in Table 2. Although the specificities of the two biomarkers were same, the sensitivity and accuracy of anti-POSTN were higher than those of anti-TIMP1. In terms of the diagnostic test, when the positive likelihood ratio was larger and the negative likelihood ratio was smaller, the diagnostic effect of biomarkers was better. Table 2 indicates that the diagnostic value of anti-POSTN autoantibody was more excellent than that of anti-TIMP1 autoantibody. All in all, the diagnosis effect of anti-POSTN for ESCC was better.

**TABLE 2:** Diagnostic value of anti-POSTN and anti-TIMP1 autoantibodies in diagnosing ESCC patients.

	AUC	95% CI	Se (%)	Sp (%)	YI	+LR	−LR	PPV (%)	NPV (%)	Accuracy (%)
POSTN	0.638	0.582–0.694	27.57	90.27	0.18	2.83	0.80	73.91	44.52	59.00
TIMP1	0.585	0.527–0.643	20.54	90.27	0.11	2.11	0.88	67.86	46.82	55.00

Se, sensitivity; Sp, specificity; AUC, area under the receiver operating characteristic curve; CI, confidence interval; PPV, positive predictive value; NPV, negative predictive value; +LR, positive likelihood ratio; −LR, negative likelihood ratio.

### The Values of Anti-POSTN and Anti-TIMP1 Autoantibodies in Diagnosing Esophageal Squamous Cell Carcinoma Patients of Different Clinical Features

The positive rate of autoantibodies in patients was calculated by taking the mean value of autoantibodies in normal controls plus the expression level of standard deviation as the cutoff value. The diagnostic values of anti-POSTN and anti-TIMP1 autoantibodies in ESCC patients of different clinicopathological characteristics, including lymphatic metastasis, distance metastasis, differentiation, TNM stage, family tumor history, gender, and age, were further explored.

**FIGURE 5:**
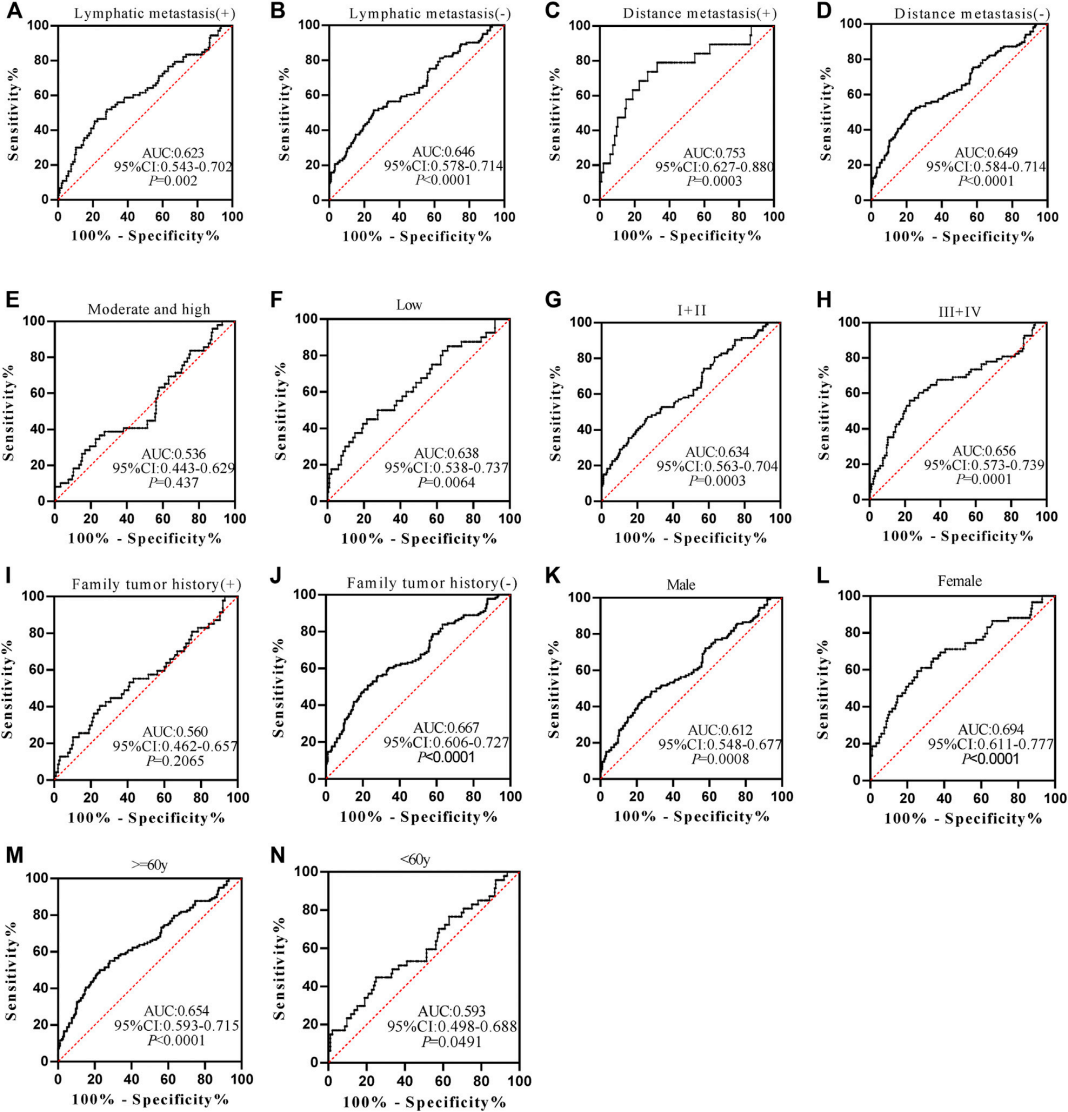
The performance of anti-POSTN autoantibody in ESCC patients with different clinical characteristics.

**FIGURE 6:**
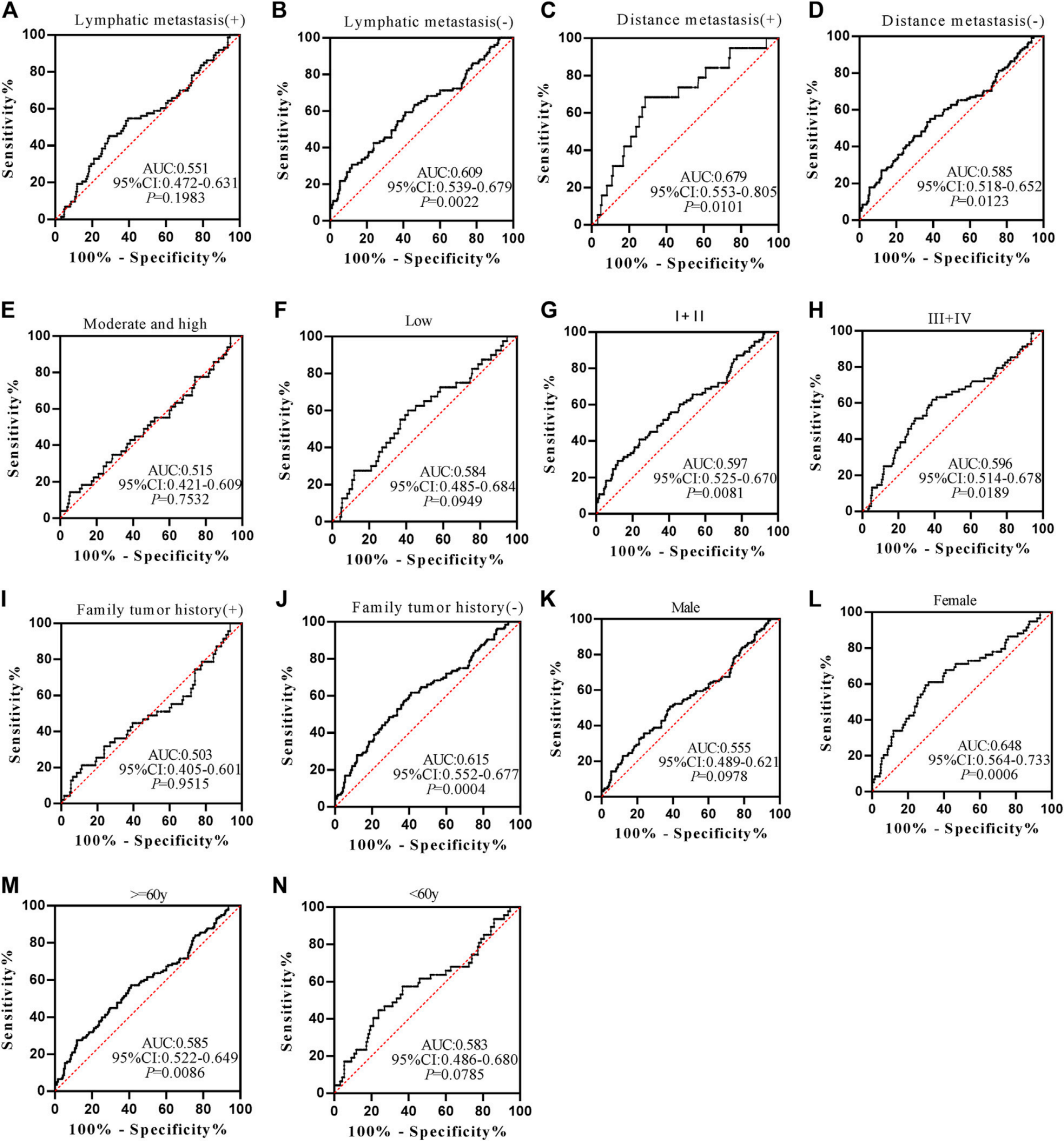
The value of anti-TIMP1 autoantibody in diagnosing ESCC patients of different clinical subgroups.

Anti-POSTN autoantibody can diagnose ESCC patients from normal controls in most subgroups except for patients with family tumor history and moderate and high differentiation, and it showed marginal difference in diagnosing ESCC patients younger than 60 years old (*p* = 0.0491) (Figures 5A–N). The AUCs of anti-POSTN autoantibody in diagnosing ESCC patients with different clinical characteristics ranged from 0.612 to 0.753 (*p*<0.05). The minimum AUC of 0.612 was observed in male ESCC patients (Figure 5K), and the maximum AUC of 0.753 was presented in patients with distance metastasis (Figure 5C). Anti-TIMP1 autoantibody can diagnose ESCC patients from normal controls in most subgroups (*p* < 0.05), but it failed to distinguish ESCC patients from normal controls in patients with lymphatic metastasis, a history of family tumor, different degrees of differentiation, and patients younger than 60 years old as well as male patients (*p* > 0.05) (Figures 6A–N). The AUC values of anti-TIMP1 autoantibody in different clinical subgroups ranged from 0.585 to 0.679(*p*<0.05). The minimum AUC of 0.585 was observed in both patients older than 60 and patients with no distance metastasis (Figures 6D,M), and the maximum AUC of 0.679 was detected in patients with distance metastasis (Figure 6C).

The positive rates of anti-POSTN and anti-TIMP1 autoantibodies were of no difference in clinical subgroups (*p* > 0.05) (Table 3). Furthermore, the AUC values of the two autoantibodies were not statistically different in all clinical subgroups (*p* > 0.05).

**TABLE 3:** Positive frequencies of autoantibodies in subgroups.

Subgroup	Anti-POSTN		Anti-TIMP1	
	Positive rate (%)	*p*	Positive rate (%)	*p*
Lymphatic metastasis				
Positive (73)	30.14	0.723	19.18	0.087
Negative (101)	32.67		30.69	
Differentiation				
Moderate and high (49)	18.37	0.124	18.37	0.305
Low (40)	32.50		27.50	
Distance metastasis				
Positive (19)	47.37	0.324	31.58	0.575
Negative (118)	35.59		27.12	
TNM stage				
I + II (93)	31.18	0.583	29.03	0.943
III + IV(68)	35.29		25.00	
Family tumor history				
Positive (47)	23.40	0.133	21.28	0.571
Negative (136)	35.29		27.94	
Age				
≥60 years (138)	38.10	0.242	27.54	0.579
<60 years (47)	25.53		23.40	
Gender				
Male (126)	29.37	0.193	23.02	0.118
Female (59)	38.98		33.90	

## Discussions

EC is a common malignant tumor worldwide. There are no obvious clinical symptoms in the early stage of EC, and there are no serum test markers that can be used for minimally invasive detection. Recent studies have identified many types of biomarkers based on GEO and TCGA datasets. For example, Zhao et al. (2021) reported that GXYLT2 might be a potential diagnostic and prognostic marker in gastric cancer based on a comprehensive analysis. Liang et al. (2019) indicated that FKBP10 may be a potential therapeutic target for the treatment of gastric cancer *via* bioinformatics analysis and immunohistochemical verification. Yang et al. (2021) confirmed that TRIB3 was a potential prognostic marker and therapeutic target for bladder cancer through bioinformatics analysis and cell function experiment. In this study, we performed differential analysis, PPI analysis, and hub gene calculation on TCGA data and GTEx data related to EC. It was gratifying that we identified two TAAbs (POSTN and TIMP1) with a potential diagnostic value for ESCC through experiment verification. To the best of our knowledge, the association of anti-POSTN and anti-TIMP1 autoantibodies with ESCC has not been reported.

Periostin (POSTN), playing a crucial role in some biological processes, is considered to be associated with tumor progression (Kudo, 2017). In several malignant tumors, such as colorectal cancer, ovarian cancer, and hepatocellular carcinoma, high POSTN expression was confirmed to be associated with poor prognosis (Lv et al., 2013; Sung et al., 2016; Deng et al., 2019). Moreover, Jin and Yang (2019) showed that POSTN could be a promising potential diagnostic biomarker for head and neck squamous cell carcinoma. Moreover, limited studies have indicated a high POSTN expression was due to poor prognostic factors for ESCC based on immunohistochemistry (Wang et al., 2014; Lv et al., 2017; Ishibashi and Tsujimoto, 2021). Based on the aforementioned research, we proposed to assume that the autoantibody produced by the protein encoded by POSTN can be used as a marker for ESCC diagnosis. In this study, we confirmed the value of anti-POSTN autoantibody in the diagnosis of ESCC, with an AUC of 0.638.

Tissue inhibitors of metalloproteinases (TIMPs) are proverbial inhibitors of metalloproteinases and composed of four structurally related members (TIMP1, TIMP2, TIMP3, and TIMP4), associated with tumor invasion and angiogenesis (Baker et al., 2002; Jiang et al., 2002). Among four TIMPs, TIMP1 overexpression or TIMP3 silencing is considered to be associated with tumor progression (Jackson et al., 2017). In gastric cancer, Mroczko et al. (2009) demonstrated that TIMP1 expression of pre-treated serum and plasma correlated significantly with the presence of distant metastases. In addition, Kozłowski et al. (2013) reported that high levels of TIMP1 in serum were related to progression and worse prognosis of patients with EC. In the current study, we found there was no significant difference of positive rates of anti-TIMP1 autoantibody between subgroups.

However, the study still has some limitations. This study is a retrospective study, so prospective studies should be carried out to verify the results. Based on the bioinformatics analyses and experimental verification presented in this study, we concluded from these results that anti-POSTN and anti-TIMP1 autoantibodies could be considered potential diagnostic markers for ESCC. We hope that our results will benefit future studies and improve the diagnosis of ESCC patients.

## Data Availability

The original contributions presented in the study are included in the article/Supplementary Material; further inquiries can be directed to the corresponding author.
